# Proximal Fibular Osteotomy in the Management of Medial Compartment Osteoarthritis of the Knee: A Prospective Study

**DOI:** 10.7759/cureus.89751

**Published:** 2025-08-10

**Authors:** Vedant Bajaj, Tarun Khare, Ambrish K Singh, Shubham Agrawal

**Affiliations:** 1 Orthopedics, Dr. Baba Saheb Ambedkar Medical College and Hospital, New Delhi, IND

**Keywords:** medial joint space, modified oxford score, osteoarthritis, pfo, vas score

## Abstract

Background: Knee osteoarthritis (OA) is a prevalent chronic, progressive, degenerative condition in older individuals, characterized by joint pain, stiffness, and deformity. The rate of articular degeneration in weight-bearing areas exceeds that in non-weight-bearing regions of the joint, which subsequently calcify to form osteophytes. Patients who do not find relief from oral and topical nonsteroidal anti-inflammatory drugs (NSAIDs) and steroids may benefit from intra-articular injections of platelet-rich plasma (PRP) and surgical interventions such as proximal fibular osteotomy (PFO), high tibial osteotomy (HTO), and total knee replacement (TKR).

Aim: This study aims to evaluate the efficacy of PFO in treating medial compartment OA of the knee.

Materials and methods: A total of 21 patients with medial compartment OA who attended the orthopedics outpatient department (OPD) during the study period of two years (2019-2020) were included. Patients diagnosed with medial compartment OA underwent PFO. The outcomes of the treatment were assessed using the visual analog scale (VAS) score and the Modified Oxford Knee Score.

Results: There was an improvement in medial knee pain in nearly all patients following PFO. Improvements were noted in the medial joint space, VAS score, and Modified Oxford Knee Score.

Conclusion: The present study demonstrates that PFO effectively alleviates pain and enhances joint function in patients with medial compartment OA at 12 months postoperatively.

## Introduction

The most common cause of disability among the elderly is osteoarthritis (OA). Pain and mobility limitations lead to disability. The primary surgical option for this patient group is total knee arthroplasty (TKA), which aims to reduce pain and improve joint function and mobility. However, TKA is expensive and complex, and after the initial procedure, some patients may require a second knee revision. Complications from the somewhat intricate high tibial osteotomy (HTO) technique include nonunion, iatrogenic fracture, and neurovascular damage. Although total knee replacement enhances function, reduces pain, and corrects alignment, it is not the preferred option for younger patients with moderate OA [1]. The surgical outcome of proximal fibular osteotomy (PFO) in medial compartment OA depends on the severity of the tibiofibular joint OA and shows a linear correlation with the patient’s BMI, the angle of the proximal tibiofibular articulation, and preoperative knee function [2]. When comparing HTO and PFO, PFO also yields excellent results [3]. Comparison of various factors between PFO and HTO for medial compartment OA [4]. Large cancellous bones compress and deform with aging and repeated pressure. There is only one cortical support in the medial upper tibial region, while there are three in the lateral aspect, with the fibula contributing two of the cortices. It is therefore logical to assume that removing a portion of this strut will relieve compression on the medial side [5]. This new procedure is cost-effective, safe, and easy to perform. Almost all patients experience pain relief following surgery. In a subset of individuals with knee OA, PFO may be utilized instead of or in addition to TKA. The current study comprehensively evaluated the short-term efficacy of PFO in alleviating pain and improving joint function among a cohort of patients at our institution.

## Materials and methods

A total of 21 patients (15 females and six males) with medial compartment OA of the knee were included in the study. The research was conducted at our tertiary medical center from July 2019 to December 2020, focusing on patients who were thoroughly examined after admission to assess their overall physical condition.

Inclusion criteria

The patients with mild to moderate symptomatic medial compartment OA (Kellgren-Lawrence (KL) classification grades 1, 2, and 3) not responding to adequate conservative treatment of the knee, with radiological evidence of medial joint space reduction.

Exclusion criteria

Patients with posttraumatic knee OA or inflammatory joint disease, those not willing to participate, those with previous knee surgery and fractures of the femur and tibia, those with Genu valgus deformity, and those patients unfit for surgery and anesthesia were excluded from the study. 

The tibiofemoral angle (TFA) and joint space were evaluated using anteroposterior and lateral X-rays of the knee in a standing position. A full-length scanogram of the whole lower limb could not be obtained because this facility was unavailable at our institution. All patients underwent routine investigations, including BMI, blood and urine tests, serum urea, creatinine, electrolytes, fasting and postprandial blood sugar, ECG, and chest X-ray. Prophylactic antibiotics in the form of parenteral third-generation cephalosporins were administered alongside the induction of anesthesia and continued for two days postoperatively.

Operative procedure

**Figure 1:**
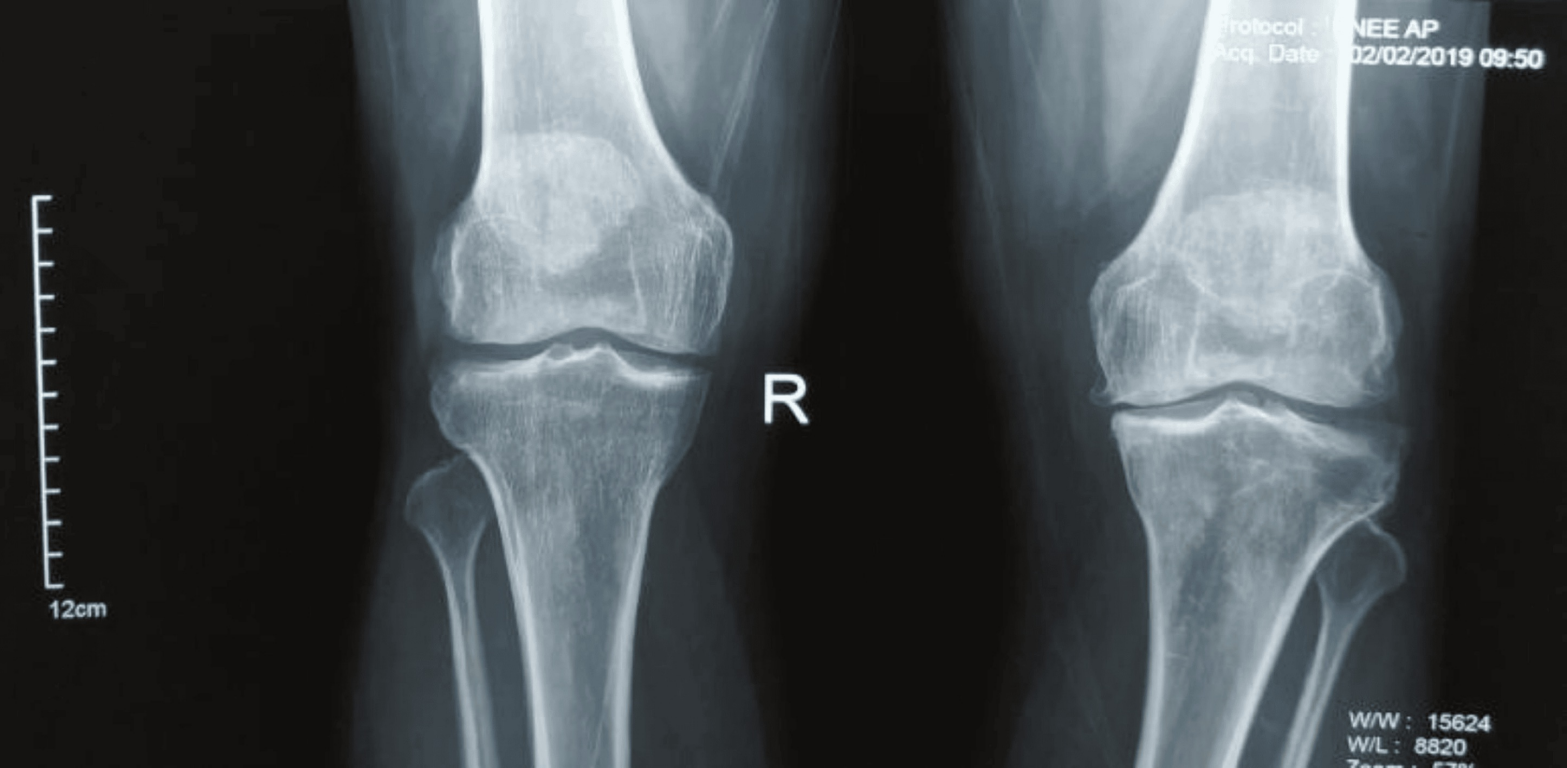
Preoperative radiograph of the patient with osteoarthritis of the right knee

**Figure 2:**
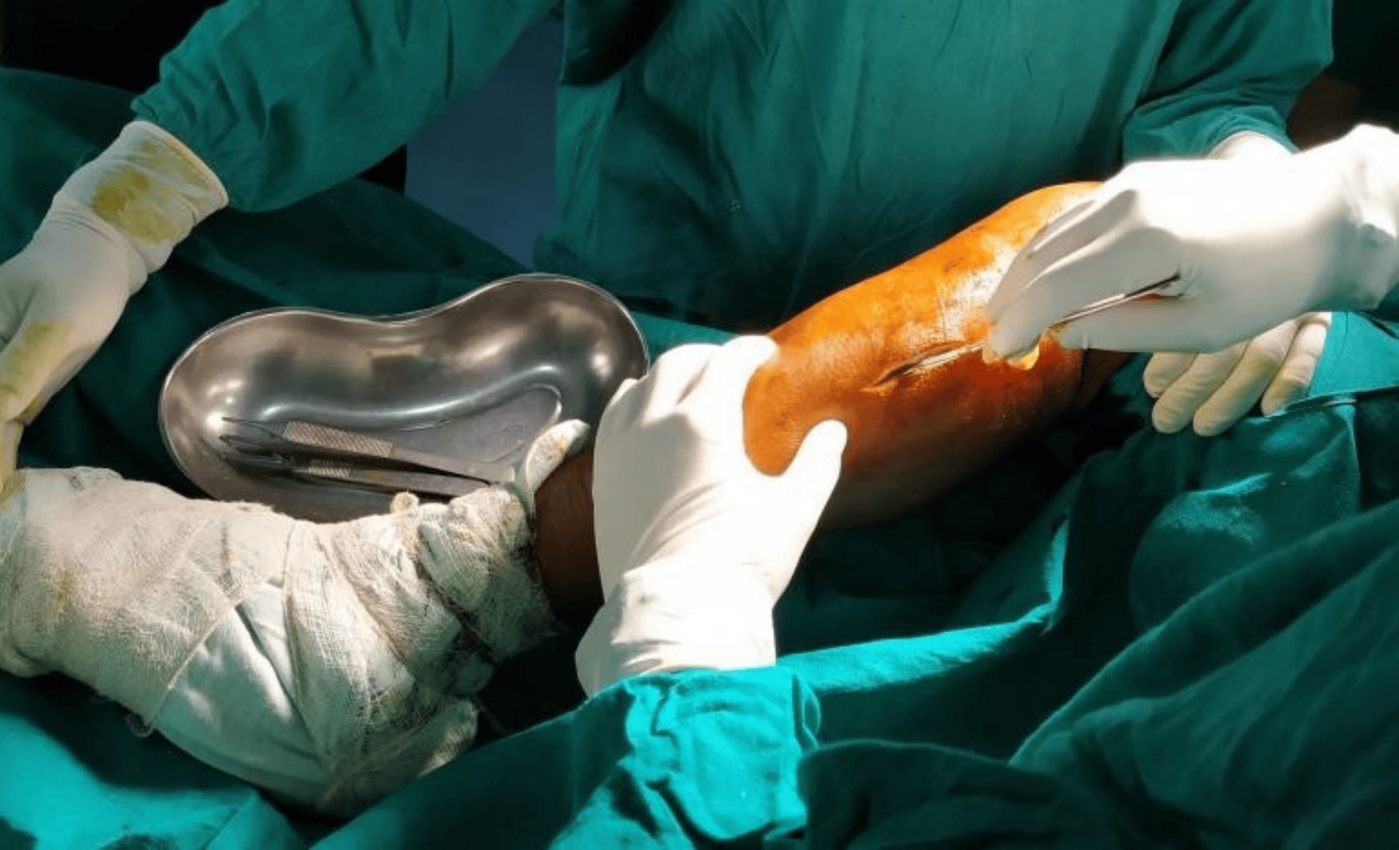
Skin incision over the proximal part of the fibula over the lateral aspect

**Figure 3:**
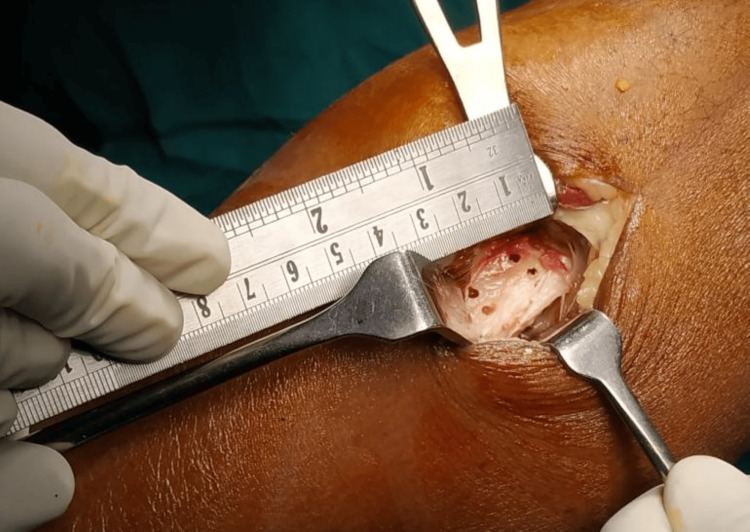
Marking confirmed with the help of measuring scale for fibula resection

**Figure 4:**
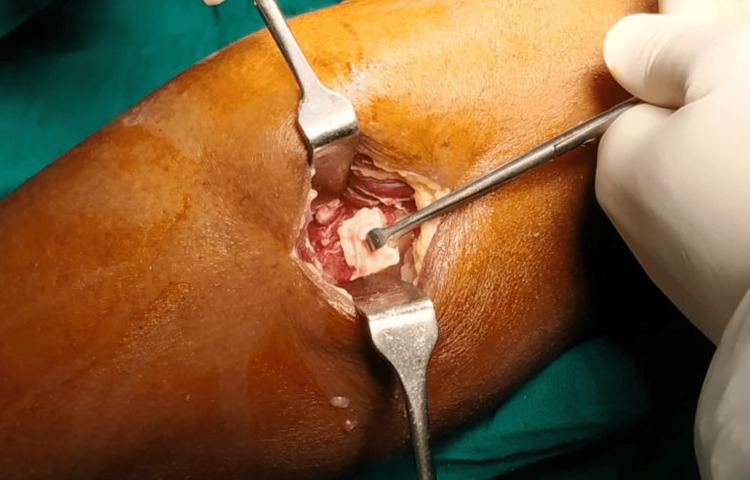
A 1.5-2 cm piece of fibula resection done

**Figure 5:**
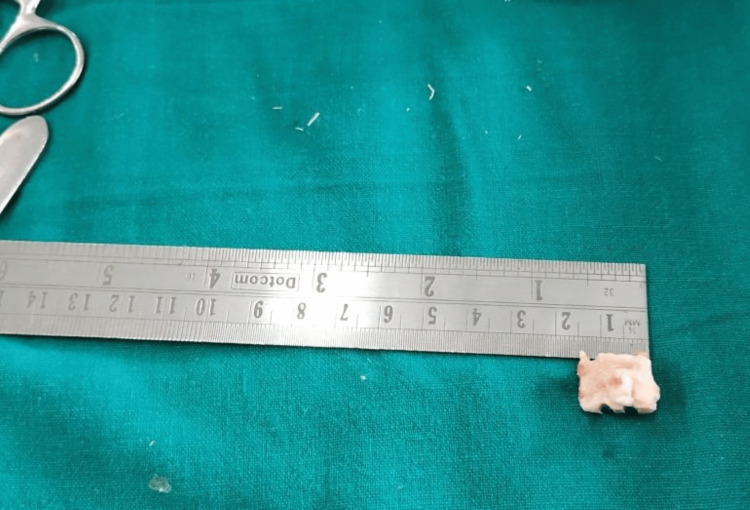
Fibular piece of bone resected and confirmed with the measuring scale

**Figure 6:**
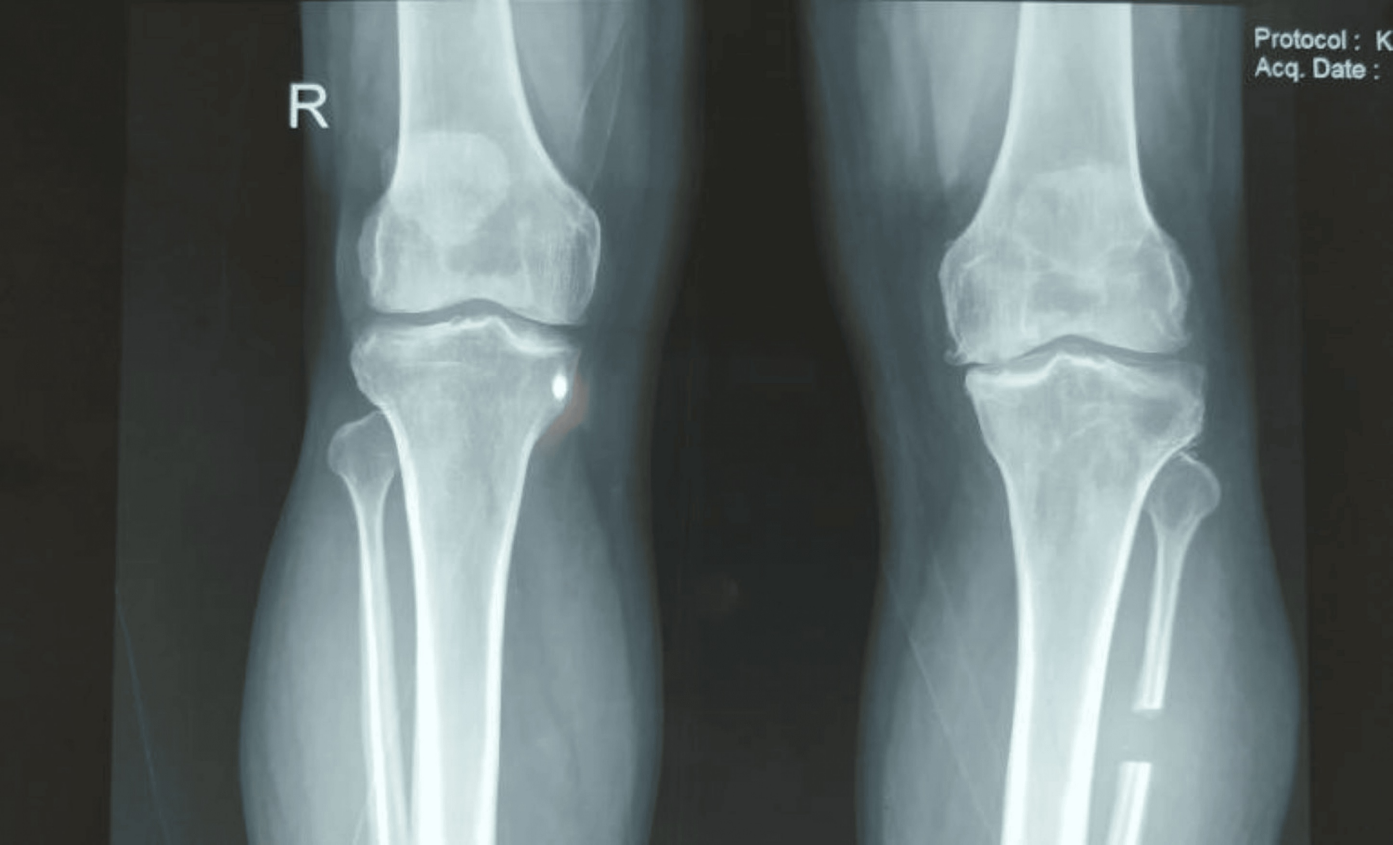
Postoperative AP radiograph showing resection of proximal fibular segment AP: anteroposterior

Using a tourniquet, the procedure was performed while the patient was in a supine position and under spinal or epidural anesthesia. A skin marking pen was used to identify the tip of the fibular head, and the appropriate downward distance was determined. The level of osteotomy should be at the junction of the upper 24% and lower 76%. If positioned higher, there is a risk of damaging the nerve; if positioned lower, the effectiveness of the surgery may be compromised. Skin and subcutaneous tissue were dissected in layers, and the incision length needed to be slightly greater than the length of the excised segment. Once the peroneus and soleus were separated, the fibular periosteum became visible. An incision of 1.5 to 2 cm was made in the periosteum, and a narrow-blade oscillating saw was utilized to remove a 1.5 to 2 cm section of the fibula, located 6 to 9 cm from the fibular head. To facilitate mechanical axis adjustment and create a more balanced knee joint with unicortical support on both sides, the goal was to remove both fibular cortices. Although the excision was sufficiently high to alter the mechanical axis, it was not high enough to harm the lateral popliteal nerve. The patient's height determines the size of the resected segment and its distance from the fibular head. Patients of smaller stature underwent a 1.5 cm segment resection, approximately 6 cm below the fibular head, while taller patients had a 2 cm resection, around 8 to 9 cm below the fibular head. After thorough cleaning with normal saline, the wound was sutured in layers and dressed (Figures 1-6).

Parenteral antibiotics and anti-inflammatory medications were administered during the initial 48 hours postsurgery. Physical therapy, patient mobilization, and weight-bearing activities were started within the first 24-48 hours following the operation. Skin sutures were removed on the 10th day after surgery. Patients received follow-up visits weekly for the first two months, monthly until the sixth month, bimonthly until the twelfth month, and then every three months at the orthopedics OPD. Clinical and functional evaluations were conducted during these follow-ups using the TFA, joint space ratio, Modified Oxford Knee Score, and the visual analog scale (VAS) score.

## Results

During the study period, 21 patients who met the inclusion criteria underwent PFO. The highest incidence of OA occurred in the age group of 51-60 years, with a mean age of 58.14 ± 3.33, accounting for 71.4% of cases. Males represented 28.6% and females 71.4%, with a gender ratio of 1:2.5. The majority of patients, 57.14%, were drivers by occupation. Right knee involvement was present in 66.7% of cases, with all 21 cases affected unilaterally. Regarding body weight, 52.4% were overweight, 33.3% were obese, and 14.3% had a normal BMI. Symptoms lasted between four and six years in 71.4% of patients, while 14.3% experienced symptoms for 1-3 years and 6-10 years. Varus deformity was observed as follows: 47.6% had 7-10°, 19% had 4-6°, 14.3% had less than 3°, and 19% had more than 10° (Table 1). A total of 33.3% of the cases were discharged within a week, and 66.7% were discharged after a week postoperatively. In our study, four patients exhibited extensor hallucis longus (EHL) weakness, and two patients experienced tingling and numbness over the dorsum of the foot postoperatively, which gradually resolved within four to six months (Table 2).

**Table 1:** Varus deformity (in degrees)-frequency distribution of patients

Varus deformity (in degrees)	No. of patients	%	Mean ± SD
1-3	3	14.3	7.90 ± 3.40
4-6	4	19.0	
7-10	10	47.6	
>10	4	19.0	
Total	21	100.0	

**Table 2:** Postoperative complications EHL: extensor hallucis longus

Postoperative complications	No. of patients	%
Absent	15	71.4
Present	6	28.6
EHL weakness	4	19.0
Tingling and numbness over dorsum of foot	2	9.5
Total	21	100.0

Comparison of outcome variables

Tibiofemoral Angle (In Degrees)

The mean TFA measured preoperatively was 182.24 ± 0.83°, while the postoperative mean was 181.43 ± 0.93° at the second month. At the sixth-month follow-up, the mean angle was 179.67 ± 0.86, and at the 12th month, it was 180.1 ± 0.77. This change was statistically significant, with a p-value of less than 0.001 (Table 3).

**Table 3:** Preop versus postop tibiofemoral angle: comparison of tibiofemoral angle at various study points from preoperative of patients studied

Variables	Min-max	Mean ± SD	Difference	95% confidence interval of the difference		t value	p-value
				Lower	Upper		
Preoperatively	181-184	182.24 ± 0.83	-	-	-	-	-
Postoperative at 2nd month	180-183	181.43 ± 0.93	0.81	0.63	0.99	9.22	<0.001**
Postoperative at 6th month	178-181	179.67 ± 0.86	2.57	2.23	2.91	15.79	<0.001**
Postoperative at 12th month	179-181	180.1 ± 0.77	2.14	1.78	2.50	12.39	<0.001**

Joint Space Ratio

The mean preoperative joint space ratio was 0.17 ± 0.01. At the second, sixth, and 12th month intervals, the mean joint space ratios were 0.18 ± 0.01, 0.2 ± 0.01, and 0.2 ± 0.01, respectively, all of which were statistically significant with a p-value of < 0.001 (Table 4).

**Table 4:** Preop versus postop joint space ratio: comparison of joint space ratio at various study points from preoperative of patients studied

Variables	Min-max	Mean ± SD	Difference	95% confidence interval of the difference		t value	p-value
				Lower	Upper		
Preoperatively	0.16-0.18	0.17 ± 0.01	-	-	-	-	-
Postoperative at 2nd month	0.17-0.19	0.18 ± 0.01	-0.01	-0.02	-0.01	-5.32	<0.001**
Postoperative at 6th month	0.19-0.21	0.2 ± 0.01	-0.03	-0.03	-0.03	-15.17	<0.001**
Postoperative at 12th month	0.19-0.21	0.2 ± 0.01	-0.03	-0.04	-0.03	-15.81	<0.001**

Modified Oxford Knee Score

The mean Oxford knee score was recorded 49.1 ± 2.95 preoperatively, and the mean Oxford knee score recorded postoperatively at the second, sixth, and 12th months were 58.67 ± 2.87, 63.14 ± 1.42, and 66.33 ± 1.98, respectively, whose p-value (<0.001) was statistically significant (Table 5).

**Table 5:** Preop versus postop Modified Oxford Knee Score: comparison of Modified Oxford Knee Score at various study points from preoperative of patients studied

Variables	Min-max	Mean ± SD	Difference	95% confidence interval of the difference		t value	p-value
				Lower	Upper		
Preoperatively	45-55	49.1 ± 2.95	-	-	-	-	-
Postoperative at 2nd month	55-65	58.67 ± 2.89	-9.57	-10.10	-9.04	-37.65	<0.001**
Postoperative at 6th month	61-65	63.14 ± 1.42	-14.05	-15.75	-12.34	-17.18	<0.001**
Postoperative at 12th month	63-71	66.33 ± 1.98	-17.24	-18.70	-15.78	-24.63	<0.001**

Visual Analog Scale

The mean VAS score was recorded 6.52 ± 0.87 preoperatively, and the mean VAS scores recorded at the second, sixth, and 12th months were 5.48 ± 0.75, 3.81 ± 0.87, and 3.14 ± 0.65, respectively, whose p-value (<0.001) was statistically significant (Table 6).

**Table 6:** Preop versus postop VAS scores: comparison of VAS score at various study points from preoperative of patients studied VAS: visual analog scale

Variables	Min-max	Mean ± SD	Difference	95% confidence interval of the difference		t value	p-value
				Lower	Upper		
Preoperatively	5-8	6.52 ± 0.87	-	-	-	-	-
Postoperative at 2nd month	4-6	5.48 ± 0.75	1.05	0.78	1.32	8.14	<0.001**
Postoperative at 6th month	3-5	3.81 ± 0.87	2.71	2.23	3.19	11.78	<0.001**
Postoperative at 12th month	2-4	3.14 ± 0.65	3.38	2.94	3.82	15.92	<0.001**

## Discussion

We compared our results with the earlier studies. We evaluated variables such as patient age, gender, occupation, BMI, symptom duration, and the affected knee side. Clinical and radiological parameters assessed included the degree of varus deformity, tibiofemoral angle, and joint space ratio. Functional assessment utilized the Modified Oxford Knee Score and VAS for pain. We also examined postoperative complications, including limb edema, EHL weakness, dorsum of foot paresthesia, and infection rates.

The mean age at presentation in our study was 58.14 ± 3.33 years, ranging from 50 to 65 years. This is younger than the subjects in other studies: Yang et al. reported 63.5 years [6], Wang et al. had a mean age of 63.96 years [7], Liu's study showed 59.45 years [8], Prakash's research found 56.3 years [9], Nie et al. observed 60.34 years [10], Zou et al. recorded 62.3 years [11], while only Subash et al. reported a younger mean age of 48.4 years [12] compared to ours. In the majority of studies, female patients were more affected. In our study, the sex ratio of male to female was 1:2.5, which is consistent with other studies. Yang et al. reported a ratio of 1:2.2 [6], Wang et al. had 1:2.9 [7], Liu had 1:5.5 [8], Prakash had 1:1.4 [9], Nie et al. had 1:4.3 [10], and Zou et al. had 1:2.3 [11], all showing a higher incidence in female patients. The average duration of symptoms in our patients was 5.04 ± 1.46 years, ranging from two to eight years. This contrasts with the study by Zou et al., where the average duration was 1.5 ± 0.4 years [11], and Yang et al., where symptom duration varied from 19 to 82 months [6]. The longer duration in our study is attributed to the patients not responding to conservative treatment for over a year before agreeing to surgical intervention. In our study, the right knee was commonly affected, with an incidence of 66.7%, similar to the findings of Prakash and Subash et al., whose studies also reported predominant right knee involvement [9,12]. The majority of patients were overweight, which aligns with the study by Subash et al., which included only patients with a BMI < 30 [12]. A high BMI with increased loading may adversely affect the healing of articular cartilage. The mean varus angle in our patients was 7.90 ± 3.40 degrees, ranging from 2° to 11°. Most (47.6%) of our cases had a varus deformity between 7° and 10°. The study subjects of Subash et al. had a varus deformity of fewer than 10 degrees [12]. Our study, as well as others, excluded severe varus knees due to the inability of PFO to correct significant malalignment. For cases with a severe degree of deformity, HTO is considered a better option. The average hospital stay duration was 8.47 ± 2.76 days, ranging from two to 11 days. Patients in our study were discharged after achieving full weight-bearing capacity, which was initiated as soon as postoperative pain allowed. In our study, the mean preoperative TFA was 182.24 ± 0.83. Follow-up measurements at the second, sixth, and 12th months showed mean TFAs of 181.43 ± 0.93, 179.67 ± 0.86, and 180.1 ± 0.77, respectively. Comparing with other studies that used TFA to measure correction of mechanical alignment, Yang et al.'s study showed an improvement in mean TFA from 182.7 ± 2.0 preoperatively to 179.4 ± 1.8 [6]. Zou et al. observed a significant improvement in TFA from 183.4 ± 2.5 preoperatively to 168.9 ± 1.3 postoperatively [11]. Similarly, Subash et al. reported an improvement in mean TFA from 182 ± 1.8 to 179 ± 1.9 [12]. Our study revealed that the mean medial to lateral joint space ratio preoperatively was 0.17 ± 0.01, which improved to 0.18 ± 0.01, 0.2 ± 0.01, and 0.2 ± 0.01 at the second, sixth, and 12th-month follow-ups, respectively, with statistical significance. Comparing our findings with Wang et al., they observed a significant increase in the joint space ratio from 0.40 ± 0.28 to 0.58 ± 0.30 at the 12-month follow-up [7]. Subash et al.'s study indicated a mean medial joint space expansion from 1.3 ± 0.8 to 4.2 ± 2.7 at the 24-month final follow-up [12]. In a similar vein, Yang et al. reported a significant decrease in lateral joint space from 7.6 ± 1.2 to 5.4 ± 1.3, focusing solely on the width of lateral joint space, which diminished from 12.2 ± 1.1 preoperatively to 6.9 ± 0.7 at the 24-month final follow-up, which was statistically significant [6]. In our study, the mean preoperative knee score was 49.1 ± 2.95, which postoperatively improved to 58.67 ± 2.89, 63.14 ± 1.42, and 66.33 ± 1.98 at the second, sixth, and 12th months, respectively, and this was statistically significant. In comparison, Prakash's study reported a mean functional knee score of 54.4 preoperatively, which increased to 77.0 postoperatively (p-value < 0.05) [9]. Similarly, Subash et al. reported a preoperative mean score of 52.2, which increased to 79.0 postoperatively (p-value < 0.05), also statistically significant [12]. In our study, all patients experienced pain as a primary clinical symptom unresponsive to conservative treatment. OA knee pain leads to functional and mobility impairments, diminishing the quality of life in the elderly. The average VAS score for pain was 6.52 ± 0.87. Postoperative assessments at two, six, and 12 months showed significant improvements in mean VAS scores of 5.48 ± 0.75, 3.81 ± 0.87, and 3.14 ± 0.65, respectively. These mean VAS scores are in line with other studies: Yang et al. reported a preoperative VAS score of 7 [6], Wang et al. had 8.02 [7], Prakash had 6.7 [9], Nie et al. had 5.64 [10], Zou et al. had 4.6 [11], and Subash et al. had 6.9 [12]. Postoperative results from these studies showed significant improvements: Yang et al. reported a VAS of 2 [6], Wang et al. had 2.74 [7], Prakash L had 2.2 [9], Nie et al. had 0.27 [10], Zou et al. had 0.5 [11], and Subash et al. had 2.1 [12]. Retractor misplacement can lead to common peroneal nerve injury, which may cause postoperative paresthesia over the dorsum of the foot, as observed in two cases from our study. Yang et al. encountered similar issues, with four (3.6%) cases of foot paresthesia and two (1.8%) cases of confirmed superficial peroneal nerve palsy [6], and Subash reported it in three (10%) of their cases [12]. The paresthesia resolved spontaneously within 4-6 months without intervention, suggesting that the nerve injuries were likely neuropraxia or axonotmesis. Zou et al. reported one case (2.5%) of neurovascular injury [11]. In our study, we encountered four cases of EHL weakness, which may have resulted from traction injury to the common peroneal nerve. To mitigate this risk, we made incisions 6 cm below the fibular head and performed osteotomies 8 cm below it. Cadaveric studies by Baruah et al. on the Northeast Indian population reported a mean fibular length of 36.1 ± 1.2 cm, with the common peroneal nerve winding anteriorly 3.2 ± 0.8 cm from the fibular head's apex [13]. Similarly, Ryan et al. found the mean fibular length to be 35.7 ± 2.1 cm [14]. Baruah et al. observed that the CPN runs superficially along the fibula's lateral surface and enters the fibular tunnel about 2 cm distal to the fibular head, wrapping around the fibular neck and broadly fanning out into three major divisions: anterior, recurrent, superficial, and deep branches [13].

## Conclusions

PFO is a dependable procedure for early-stage OA of the knee with genu varum deformity, provided the deformity is less than 15°. The effectiveness of PFO largely stems from the release of tight soft-tissue structures connected to the fibular head and the correction of uneven loading on the tibial weight-bearing articular surface. PFO is a straightforward and quick operation that can be completed within 30-40 minutes with minimal blood loss, approximately 30-50 ml. The procedure allows for early ambulation with full weight-bearing in the postoperative period, which is an additional benefit. Early functional and radiological outcomes have shown promise in terms of pain relief, improved functional scores, correction of the tibiofemoral angle, and a slight increase in medial joint space. Our hypothesis that PFO alleviates pain and improves the functional status of patients by shifting the mechanical axis from the medial to the lateral compartment and reducing pressure in the medial compartment appears valid, although the precise mechanism requires further study. Postoperative complications such as paresthesia over the dorsum of the foot and EHL weakness can be prevented by careful retractor placement and avoiding excessive stretching of soft tissues, which minimizes injury to the branches of the CPN. Therefore, in appropriately selected cases, PFO may serve as an alternative to HTO or unicompartmental knee arthroplasty (UKA) for early OA knees with genu varum deformity to prevent disease progression. If disease progression eventually necessitates TKA, it can be safely performed without any complications due to prior PFO. The limitations of our study were a small sample size and our inability to obtain a scanogram at our center.
